# Impact of Premature Greying of Hair on Socio-cultural Adjustment and Self-esteem among Medical Undergraduates in Foundation University, Islamabad

**DOI:** 10.7759/cureus.5083

**Published:** 2019-07-04

**Authors:** Muhammad Saad, Nabeela Fazal Babar, Ramsha Majeed, Ateeq Ur Rehman, Omair A Khan, Danyal E Chatha, Urooj Aamir, Amna Nadeem, Sayeddah Abbas

**Affiliations:** 1 Internal Medicine, Fauji Foundation Hospital, Rawalpindi, PAK; 2 Community Medicine, Foundation University Medical College, Rawalpindi, PAK; 3 Rehabilitation, Fauji Foundation Hospital, Rawalpindi, PAK; 4 Biochemistry, Fauji Foundation Hospital, Rawalpindi, PAK

**Keywords:** self-esteem, premature greying of hair, socio-cultural

## Abstract

Introduction

Greying of hair is an inevitable phenomenon that occurs commonly as a person ages. It usually occurs in the fourth decade regardless of gender but now a days, even people in their early twenties can experience premature greying. The objective of our study was to determine the frequency of premature greying of hair and its impact on the socio-cultural spheres of life and self-esteem of medical students.

Methodology

It is a descriptive cross-sectional study conducted at Foundation University Medical College (FUMC) from January to February 2017. All medical students of FUMC who gave their consent were included in the study sample. Students who were absent or unwilling to participate were excluded. Data was collected through a self-administered questionnaire, which was then analysed using SPSS version 17 (SPSS Inc., Chicago, IL).

Results

Out of 673 students, 210 (31.2%) suffered from premature greying of hair. The prevalence was higher among females (155/73.8%) as compared to males (55/26.2%). There was a statistical difference in perception of both the genders, regarding those with premature greying of hair trying to hide it (p-value = 0.000), premature greying of hair as contagious (p-value = .009) and the affected looking older than their actual age (p-value = 0.036).

Conclusion

The study showed that premature greying affected the socio-cultural spheres of students’ lives. Females were more prone to developing premature greying of hair. Genetics also play a significant role in the phenomenon. No positive correlation was found by using Rosenberg Self-esteem scale.

## Introduction

Hair greying or canities is a normal phenomenon occurring as one ages, but some individuals experience hair greying at a much earlier age. Hair contains melanin, a pigment whose production is determined by the genetic makeup of a person. Melanin gives colour to our hair, eyes and skin. The process of hair greying has been attributed to the loss of pigment forming melanocytes, which are the product of a complex biological pathway, i.e., melanogenesis [1]. Although hair greying or canities is a natural process occurring in people as they age, an unknown percentage of individuals experience premature greying from familial inheritance or pathologic conditions. It has been ascertained that grey hair does not contain any melanocytes, or it contains very few which are inactive [2]. The occurrence of pigmentation is an important phenotype of humans. The exact etiology for premature greying of hair (PGH) remains uncertain to this day but low serum calcium, serum ferritin and vitamin D3 levels have been linked to premature greying of hair [3]. A decrease in the synthesis of melanin is associated with the reduced activity of tyrosine. Free radical theory of hair greying has also been suggested [4]. PGH has been found to be associated with a number of autoimmune diseases, disorders of endocrine systems, UV radiation, drugs, smoking, deficiencies of trace elements and nutritional deficiencies. Calcium and vitamin D3 deficiencies play a less prominent role. It is an indicator for either genetic diseases or non-genetic diseases such as myocardial infarction, congestive heart failure, stroke, liver cirrhosis, or cancer [5,6].

Premature greying of hair is seen among young adults; starting in Caucasians before the age of 20 years, while in Africans before the age of 30 years. Due to an increase in its incidence more people are giving medical attention to its psychological effects [7]. Studies regarding loss of hair pigmentation and associated factors have been done in other Asian countries like Turkey and India [8,9]. The lack of studies conducted in our area of the world, however, prompted us to perform a cross-sectional study among medical students to determine the frequency of premature greying of hair (PGH), how PGH affects psychological health, socio-cultural adjustment and issues rooting from low self-esteem in medical students.

## Materials and methods

It is a descriptive cross-sectional study conducted in Foundation University Medical College (FUMC) Islamabad from January to February 2017. Target population were medical students of Foundation University Islamabad Campus. All medical students of FUMC who gave their consent were included in study sample. Students who were absent or unwilling to participate were excluded. A sample size of 446 was calculated at a 95% confidence interval and 5% sample error with a prevalence of 50%. Effect on socio-cultural adjustment was assessed by using a preformed & pretested questionnaire which was distributed in classrooms and re-collected after 15-20 minutes. Approval for the project was obtained from the local ethical committee. The questionnaire used in this study had several parts, including;

· Socio-demographic part.

· Perception of students about premature greying.

· Cause ordered on five-level Likert Scale (Strongly agree, Agree, Neutral, Disagree, Strongly disagree).

· Social and cultural impact of premature greying of hair in both the genders.

The impact on self-esteem was calculated by using Rosenberg Self-esteem Scale. Simple tables were made for each variable, showing the results in numbers and percentages. Data analysis was done by using SPSS version 17 (SPSS Inc., Chicago, IL). Descriptive statistics including means, standard deviations, and frequencies were assessed on variables. Chi-square test was used for quantitative variables.

## Results

The study sample consisted of 673 medical students from first year to final year. Mean age of the students was 20.94 yr ± 1.85 standard deviation. Most of the students, i.e. 490 (72.8%) students, were between the ages of 19-22 year whereas between 17-18 year were 46 (6.8%) and 23-26 were 137 (20.3%) students. Refer to Table 1.

**Table 1:** Age of study participants

	Age	n
1.	17-18	46
2.	19-20	265
3.	21-22	225
4.	23-24	107
5.	25-26	30
	Mean Age	20.94 ± 1.85

Out of 673 students, 500 (74.3%) were females and 173 (25.7%) were males. It showed female predominance.

A total of 210 (31.2%) study participants have experienced premature greying of hair. Significant number of participants had reported early greying of hair. See Table 2.

**Table 2:** Prevalence of premature greying of hair

Are you affected by premature greying of hair?	n (%)
Yes	210 (31.2%)
No	463 (68.8%)

Overall prevalence was 210 (31.2%) out of 673 students participated. Third year had maximum reporting of premature greying of hair whereas 5th year had least number of students affected. Females were affected more. See Table 3.

**Table 3:** Class- and gender-wise distribution

Year	Male	Female	Total n (%)
Prevalence in 1^st^ year	10	29	39 (18.57%)
Prevalence in 2^nd^ year	11	33	44 (20.95%)
Prevalence in 3^rd^ year	13	34	47 (22.38%)
Prevalence in 4^th^ year	13	32	45 (21.42%)
Prevalence in 5^th^ year	8	27	35 (16.66%)
			Total = 210 (100%)

When asked, students strongly agreed that genetics, malnutrition and erratic lifestyle played an important role in the onset of premature greying of hair. Junk food, smoking, obesity and blood donation were not a risk factor for premature greying of hair. See Table 4.

**Table 4:** Factors responsible for causing premature greying of hair

Factors responsible for premature greying of hair		Strongly disagree	Disagree	Neutral	Agree	Strongly agree
1	Genetics	70 (10.4%)	39 (5.79%)	45 (6.68%)	206 (30.60)	313 (46.50%)
2	Junk food	142 (21.09%)	176 (26.15%)	195 (28.97%)	105 (15.60%)	55 (8.17%)
3	Smoking	78 (11.58%)	149 (22.13%)	201 (29.86%)	167 (24.81%)	78 (11.58%)
4	Obesity	125 (18.57%)	198 (29.42%)	212 (31.50%)	89 (13.22%)	49 (7.28%)
5	Malnutrition	75 (11.14%)	83 (12.33%)	125 (18.57%)	232 (34.47%)	158 (23.47%)
6	Erratic lifestyle	72 (10.69%)	111 (16.49%)	196 (29.12%)	200 (29.71%)	94 (9.50%)
7	Blood donation	239 (35.51%)	151 (22.43%)	164 (24.36%)	68 (10.10%)	51 (7.57%)

When the students were asked when they considered greying of hair as premature, most of the students, i.e. 257 (38.18%), believed it started around the age of 16-20. Students believed that premature greying of hair was not associated with a lack of self-care. However, a sizeable portion believed that it might be associated with the use of excessive cosmetic hair products and inadequate oiling. Refer to Table 5 for more details.

**Table 5:** Perception about age of greying as premature and self-care related

	Options, n%							
When do you consider greying of hair as premature?	10-15 years	16-20 years		21-25 years		26-30 years		More than 30
	151 (22.44%)	257 (38.19%)		133 (19.7%)		90 (13.3%)		42 (6.24%)
Is it associated with self-care?	Inadequate oiling of scalp		Use of cosmetic/hair products		Inadequate washing		Not associated with lack of self-care	
	95 (14.1%)		178 (26.4%)		25 (3.7%)		375 (55.7%)	

When asked, the affected students reported noticing greying of hair for the first time around the age of 16-20 years. The students mostly used different styling techniques to hide their grey hair as well as dying techniques. Anxiety, mood fluctuation, concentration at work, interpersonal relationship and treatment-related questions did not reveal any positive responses. Fifty-seven (27.1%) students believed that they looked older than their actual age while many denied this. Refer to Table 6 for more details.

**Table 6:** Psychological impact on students affected by premature greying of hair in study population (n = 210) PGH: Premature greying of hair

Sr. No	Variable	Frequency	Percentage
1	First noticed PGH at the Age of?		
	Less than 16 years	47	22.38%
	16-20 years	116	55.23%
	21-25 years	30	14.28%
	Other	17	8.09%
2	How do you hide your grey hair?		
	Dyeing	59	28.09%
	Styling	93	44.28%
	Wear cap	11	5.23%
	Covering	47	22.38%
3	Felt guilty/anxiety about having grey hair?		
	No	147	70%
	Yes	63	30%
4	Fluctuations in mood due to grey hair?		
	No	177	84.28%
	Yes	33	15.71%
5	Does PGH affect your concentration at work?		
	No	174	85.85%
	Yes	36	17.14%
6	Does PGH affect your interpersonal relationship?		
	No	173	82.38%
	Yes	37	17.61%
7	Feel the need for treatment?		
	No	139	66.19%
	Yes	71	33.81%

As depicted in the following Table 7, there was a statistical difference in perception of both the genders regarding, those with premature greying of hair try to hide it (p-value = 0.000), premature greying of hair is contagious (p-value = .009) whereas no statistically difference was found regarding their opinion on how frequently greying of hair is noticed (p-value = .011), if premature greying of hair is a sign of old age and loss of health (p-value = 0.215) and premature greying of hair hinders one from participating in social activities (p-value = 0.115).

**Table 7:** Gender-wise difference in perception to premature greying of hair in study population (n = 673) PGH: Premature greying of hair

Sr. No.	Variable	Gender		P-value
1	People with PGH are frequently noticed and commented upon by others?	Male	Female	0.113
		No. %	No. %	
	No	58 (8.61%)	136 (20.20%)	
	Yes	115 (17.08%)	364 (54.08%)	
2	People with PGH try to hide it?			0.000
	No	62 (9.21%)	107 (15.89%)	
	Yes	111 (16.49%)	393 (58.39%)	
3	PGH is contagious?			0.009
	No	141 (20.95%)	450 (66.86%)	
	Yes	33 (4.90%)	49 (7.28%)	
4	PGH is a sign of old age and loss of health?			0.215
	No	62 (9.21%)	107 (15.89%)	
	Yes	111 (16.49%)	393 (58.39%)	
5	PGH hinders from participating in social activities?			0.115
	No	117 (17.38%)	369 (54.82%)	
	Yes	56 (8.32%)	130 (19.31%)	

As seen in Table 8, there was a statistical difference in psychological impact on both genders who were affected by premature greying of hair, those with premature greying of hair look older than their actual age (p-value = 0.036), whereas no statistical difference was found regarding if they feel guilty/anxiety (p-value = 0.304), mood fluctuations due to premature greying of hair (p-value = 0.135), premature greying of hair affects your concentration at work (p-value = 0.678), premature greying of hair affects your interpersonal relationship (p-value = 0.410) and if you have tried any treatment for premature greying of hair (p-value = 0.267).

**Table 8:** Gender-wise difference in psychological impact on students affected by premature greying of hair in study population (n = 210) PGH: Premature greying of hair

Sr. No.	Variable	Gender		P-value
1	Felt guilty/anxiety about having grey hair?	Male	Female	0.304
		No. %	No. %	
	No	42 (20%)	105 (50%)	
	Yes	13 (6.19%)	50 (23.8%)	
2	Fluctuations in mood due to grey hair?			0.135
	No	50 (23.8%)	127 (60.4%)	
	Yes	5 (2.3%)	28 (13.3%)	
3	Does PGH affect your concentration at work?			0.678
	No	47 (22.3%)	127 (60.4%)	
	Yes	8 (3.8%)	28 (13.3%)	
4	Does PGH affect your interpersonal relationship?			0.410
	No	43 (20.4%)	130 (61.9%)	
	Yes	12 (5.7%)	25 (11.9%)	
5	Tried any treatment?			0.267
	No	44 (20.9%)	135 (64.2%)	
	Yes	11 (5.2%)	20 (9.5%)	
6	People think you are much older than your actual age?			0.036
	No	34 (16.1%)	119 (56.6%)	
	Yes	21 (10%)	36 (17.1%)	

To calculate the self-esteem of the students, traditional Rosenberg Self-Esteem Scale (RSES) was used. This scale has been used traditionally to estimate self-esteem. In this scale, 10 questions were used with a maximum score of 30. Score below 15 indicates low self-esteem. All students were allowed to use this tool. Students were divided into two categories, i.e., students affected and students not affected by premature greying of hair. Those students who were affected by premature greying of hair yielded a mean score of 20.27 whereas students who were not affected by premature greying of hair yielded a mean score of 19.3. No positive correlation was found between low self-esteem and premature greying of hair. Moreover, it yielded a p-value of 0.23 which is statistically insignificant. See Table 9.

**Table 9:** Rosenberg self-esteem scale PGH: Premature greying of hair; RSES: Rosenberg self-esteem scale.

Mean score using RSES	
Students affected by PGH	20.27
Students not affected by PGH	19.3

## Discussion

A study was conducted on the medical students of Foundation University Medical College. The aim of this study was to assess the general awareness regarding PGH and the common opinions regarding causes of premature greying, as well as its psychological and socio-cultural effects on students.

Canities is a natural phenomenon commonly associated with the process of human aging. Hair plays a major role in our overall appearance especially in women as they tend to base their dignity on their appearance. Premature canities are becoming increasingly prevalent, leading to issues of low self-esteem, poor quality of life and poor health. People suffering from PGH think that they are often noticed because of their imperfect hair, leading to a feeling of low self-esteem [10].

Less than one-third (31.2%) of our respondents reported to experiencing premature greying of hair. A notable finding of our research was that it was more prevalent in females as compared to males. In our study, it was revealed that 73.8% of the affected students were females. Whereas according to the case control study conducted in 2016-2017 in North India, it was found that males are more affected by PGH, i.e., 55% [11]. This high percentage in our setting can be justified by considering the fact that male to female ratio in our study was more inclined towards female gender (74.3% females and 25.7% males). When asked at what age they consider greying of hair to be premature, a majority of students considered the ages of 16-20 years as premature, while only a few considered the age of PGH to be above 30. This shows adequate general knowledge of the students. More than half of the students said that people with PGH were often noticed and commented upon by others. This finding has been supported by a study conducted by The University of Delhi in Northern India which reported that people with PGH had to face embarrassment as well as mockery from both peers and seniors [12]. Almost 12% of students said that they believed PGH was contagious whereas in Northern India they found that one-third of their sample population considered PGH as contagious. The reason behind this difference could be that the present study has been conducted on medical students and therefore it is more logical for them to believe that greying of hair is not contagious [12]. More than half of the students considered PGH to be indicative of old age and loss of health. Nearly three-fourths of our sample population reported that this condition did not affect their participation in social activities. More than half of the subjects of our research sample disagreed with the statement that PGH is associated with lack of self-care. Out of the remaining subjects, around one-third of the students considered use of hair products as a cause of PGH. With regards to evaluation of the factors that participate in premature greying of hair, the research conducted by Tobin on the aging of hair follicle pigmentation system stated that genetics play a role in premature greying of hair because it is inherited as an autosomal dominant trait [13]. This means that either of your close relatives, siblings or parents might have a history of premature greying of hair. First-degree relatives are at a higher risk of having premature hair greying followed by the second- and third-degree relatives. A study conducted among men under the age of 30 also suggested that an association with family history plays a role in premature greying of hair [14]. Premature greying of hair is an independent trait and does not present with any other abnormality. A majority of the respondents in this study were of the opinion that genetics play a vital role in PGH. Smoking has the tendency to cause obvious changes in the hair pigmentation leading to premature greying. The research conducted in our university showed that the majority of the students did not consider smoking as a factor that can influence premature greying. Chronic smoking was found to have a strong association with premature greying of hair as it causes impairment of the regenerative capacity of stem cells, which was supported by the studies conducted in Korea and Jordan [14,15]. The role of obesity in premature greying of hair is not well defined because only a few researches have been conducted to support this theory. The research conducted by the hospitals in Korea stated that obesity is an important factor of the phenomenon while the research conducted in our university showed that a major portion of the students did not consider obesity to be an influencing factor [14]. It was seen that malnutrition can be one of the causes of premature greying of hair. Severe loss of important nutrients like calcium, vitamin D3, iron, zinc and copper showed a significant association. It is also observed that people who suffer from premature greying of hair have a deficiency of these minerals [3,16]. Almost one-third of the students who participated in the research conducted in our university, considered malnutrition as a factor causing greying of hair.

On further analysis of the questions asked, specifically from people suffering from PGH, it was observed that more than half of them started experiencing PGH between the ages of 16-20 years. This is in accordance with another research that labelled 20 in Caucasians as the age for premature greying of hair [17]. Less than half of these individuals feel the need to hide their hair, mostly by styling. This result was supported by the North Indian research which stated that nearly three-fourths of patients tried to conceal their condition by styling and coloring [12]. When asked about the psychological effects of PGH, a majority of the individuals said that it had no effect on their anxiety or mood, and they felt no guilt. This was contradicted by the research conducted by Daulatabad et al. that reported a sense of guilt associated with lack of self-care. This same research stated that the concentration in affected individuals is preserved, which is supported by our own results [12]. The self-esteem of the respondents was evaluated by Rosenberg Self-Esteem Scale. Its effectiveness is validated worldwide as well as nationwide [18-20]. This scale has never been used previously in studies related to premature hair greying. When the self-esteem of the respondents suffering from premature hair greying was compared with those who did not experience PGH, it was observed that there was no significant difference between the two. The study failed to show an association between premature greying of hair and low self-esteem.

The study showed that premature greying affected the socio-cultural spheres of students’ lives. Females are more prone to developing premature greying of hair. Genetics, malnutrition and erratic lifestyle also play a significant role in premature greying. Statistically significant data was reported in people with premature greying of hair who try to hide it (p = 0.000) and they look older than their age (p = 0.036). No positive correlation was found by using Rosenberg Self-esteem scale.

## Conclusions

The present study concludes that premature greying of hair is mostly seen between 16-20 years of age, with females being more prone to developing premature grey hair. Genetics plays an important role in premature greying of hair. Those with premature greying often get noticed for it, causing a hindrance in their social activities. Statistical difference was observed in ‘people with premature greying of hair who try to hide it’ and ‘they look older than their age’.

## Appendices

We recommend that research on extensive population or on a larger sample size should be conducted. As this research on premature canities is one of its kind in Pakistan, further research on this topic should be greatly encouraged. As premature canities causes a hindrance in social activities, the affected individual should be encouraged to participate in more social events and provided an equal opportunity to compete.
